# The Effect of Liquid Crystalline Structures on Antiseizure Properties of Aqueous Solutions of Ethoxylated Alcohols

**DOI:** 10.3390/ijms11010189

**Published:** 2010-01-12

**Authors:** Marian Wlodzimierz Sulek, Anna Bak

**Affiliations:** Technical University of Radom, Department of Physical Chemistry, 26-600 Radom, Chrobrego 27, Poland; E-Mail: a.bak@pr.radom.pl (A.B.)

**Keywords:** liquid crystals, ethoxylated alcohol solutions, antiseizure properties

## Abstract

Aqueous solutions of ethoxylated alcohols which form lyotropic liquid crystals at high concentrations (40–80%) were selected as model lubricating substances. Microscopic studies under polarized light and viscosity measurements were carried out in order to confirm the presence of liquid crystalline structures in the case of alcohol solutions with ethoxylation degrees of 3, 5, 7 and 10. Microscopic images and viscosity coefficient values characteristic of various mesophases were obtained. As expected, the viscosity of LLCs decreases considerably with an increase in shearing rate which is characteristic of liquid crystals being non-Newtonian liquids. Antiseizure properties were determined by means of a four-ball machine (T-02 Tester) and characterized by scuffing load (P_t_), seizure load (P_oz_) and limiting pressure of seizure (p_oz_). Alcohol ethoxylates forming mesophases in aqueous solutions have the strongest effect on the P_t_ values which are several times higher than those measured in the presence of water. Ethoxylates with higher degrees of ethoxylation exhibit higher values of scuffing load. Those changes have been interpreted as a result of higher cloud points at which those compounds lose their amphiphilic properties. In general, the presence of mesophases in the bulk phase and particularly in the surface phase may lead to the formation of a lubricant film which separates the frictionally cooperating elements of a friction pair. The antiseizure efficiency of alcohol solutions is highest up to the load value which does not exceed the scuffing load value.

## 1. Introduction

The main aim of tribological research is optimization of friction processes leading to a reduction in material and energy losses and an improvement in failure-free operation of machines and devices. A lubricating substance, often called “a third body”, is an essential component of a tribological system.

Functional properties of lubricating substances can be devised at the molecular level by selecting molecules having appropriate, specific properties. Lubricating substances are usually multicomponent solutions consisting of a base and additives modifying their tribological properties. Designing a suitable composition of a lubricating substance ensures formation of a top layer under friction conditions which protects friction node materials against direct contact and adhesive wear.

The starting point for the search for a new type of lubricating substances were useful tribological properties of molybdenum disulfide and graphite which have been used as additives in various oil bases. Their influence on friction conditions has been interpreted as a result of formation of crystalline structures (hexagonal structures) in which easy slip planes appear. Aqueous solutions of ethoxylated alcohols which form lyotropic liquid crystals at high concentrations (40–80%) have been selected as potential lubricating substances. It has been assumed that they will form, both in the bulk phase and in the surface phase, mesophases which, under friction conditions, will turn into a lubricant film protecting friction pairs against seizure. Ecological considerations are an important criterion for formulation of lubricating substances. Therefore, water has been chosen as a base and alcohol ethoxylates, which are not hazardous to the natural environment, have been selected as additives.

A crystalline solid has an internal order. Its elements are arranged regularly according to geometrical rules. Apart from a geometrical order there may occur an orientational order in solids. It occurs when crystalline lattice components have a spatial structure characterized, for example, by a dipole structure. Both the geometrical order and the orientational order are of the long-range type, and are many times larger than the sizes of crystal lattice components. Solids occur in the form of monocrystals and polycrystals. A polycrystalline aggregate consists of microscopic-size crystals larger than 1 μm. The characteristic feature of the crystals is cracking along certain directions. Other properties of crystals also depend on their orientation, *i.e.,* they exhibit anisotropy.

“Liquid crystals” are an intermediate state between crystalline solids and an isotropic liquid. Therefore, it is more accurate to call them mesophases or intermediate phases. They are stable from the point of view of thermodynamics. They exhibit a long-range order and anisotropic properties characteristic of a solid. Compared to components of solids, they also exhibit higher mobility of molecules or atoms characteristic of a liquid. Liquid crystals can be divided into thermotropic and lyotropic. The subject of interest in this paper are lyotropic liquid crystals [1,2].

The basic components of structures of lyotropic liquid crystals (LLCs) are micelles. They consist of monomers whose structure differs from spherical symmetry. The shape of micelles depends both on the structure of molecules, solvent properties, concentration in solution and the presence of other solution constituents. The presence of electrolytes and also molecules of non-polar substances in the solutions is particularly important [3–8]. The ordering of micelles may lead to various spatial and orientational structures. Formation of the structures which result from long-range interactions also depends on temperature, although it is not the most important factor. Therefore, it can be stated that lyotropic mesophases are formed as a result of an interaction with a solvent and this process can also be affected by temperature.

The molecules forming micellar solutions and then liquid crystals have various structures. Particular attention should be paid to amphiphilic surface-active agents. Amphiphiles form liquid crystalline structures if micelles are solvated with a solvent. Not exceeding the temperature above which alkyl chains undergo “liquefaction” is an additional condition. Thus, the mesomorphic state occurs below the critical temperature whose value depends mainly on the alkyl chain, its length, saturation ratio and branching [9,10]. The examples of the type of amphiphiles which form a liquid crystalline state are monoglycerides and lipids being components of biomembranes. Not all surface-active agents can form mesophases. Some of them, such as di- and triglycerides, fatty acids and alcohols, sterols and certain hormones exhibit surface activity and gather at the interface but they do not form mesophases.

## 2. Results and Discussion

### 2.1. Polymorphism of Lyotropic Liquid Crystals

The structure and size of micelles as well as the type of solvent affect the possibility of forming many varieties of polymorphic LLCs whose presence has been confirmed experimentally. The type of mesophase formed depends on other components of solutions, e.g., electrolytes and on external pressure as well as electric and magnetic field temperatures [7]. LLCs may form both in polar and non-polar solvents and may consist of straight and reverse micelles. The basic polymorphic varieties include the following mesophases: nematic, hexagonal, lamellar and cubic [9,10].

The nematic phase apppears commonly in thermotropic liquid crystals. It was identified in LLCs relatively late (1967) [11] and it appears in few systems over a narrow range of concentrations and temperatures. Those structures form frequently in the presence of an electrolyte [11–13]. The nematic phase shows a lack of long-range positional order and it exhibits only orientational order. This is the simplest form of orientational order. There appears one director along which micelles, most commonly short cylinder- and disk-shaped, are aligned. This phase has a relatively low viscosity and an orientational ability resulting from external conditions such as substrate structure, electric and magnetic fields and liquid flow.

The hexagonal phase occurs over a wide range of temperatures and surfactant concentrations (30–50%) and is composed of straight and reverse micelles. It is characterized by a particularly high viscosity and, as such, it is easily distinguishable. The viscosity may be so high that the liquid does not flow. The hexagonal phase is composed of cylindrical micelles whose hydrophilic groups, in the case of straight micelles, are oriented towards a polar solvent (e.g., water). The cross-section of the micelles may be round or oval-shaped. They are predominantly long cylinders resembling threads. There also appear shorter micelles of double-diameter lengths. The diameters of the micelles are from 1.5 to 2 times larger than the length of the monomers, and the distances between the micelles are from tenths to several nanometers [3]. The hexagonal structures of LLCs display a characteristic fan texture under a polarizing microscope.

Lamellar phases appear over a wide concentration range, usually at higher concentrations than those for hexagonal phases. They are also generated at low concentrations if micelles are in the form of large bilayers [7]. The characteristic feature is a decrease in viscosity compared to hexagonal phases and that proves their presence. The viscosity drop is caused by the layer structure of this phase which results in easy slip planes.

The lamellar phase may consist of disk-shaped micelles or bilayers [14]. The latter have two planes with non-penetrating or mutually penetrating hydrocarbon chains whose thickness is of the order of 1.5–2 chain lengths. The sizes of the planes are theoretically limited by the size of the vessel. In fact, the planes are finite-size. Individual lamellar structures can be separated by a solvent, e.g., water or alkanes. Solvation and incorporation of a solvent between planes result in swelling of the structures. The thickness of the layers can be relatively high and it may reach even few nanometers in the case of dilute solutions [15].

The cubic phase exhibits a relatively high viscosity. The cubic phase constituents are mostly spherical micelles but also “deformed” spheres, short rods and disks [16]. A lattice can also be formed from a continuous bilayer of a surfactant and a continuous dispersing constituent and that is called a birefringent structure. Thus, both discrete and continuous regular lattices are composed of two kinds of aggregates. In the case of the continuous one the surfactant and the solvent form independent mutually penetrating layers. The structures built of double surfactant layers have constant spacings and constant radii of curvature [17].

The cubic phases composed of finite-size micelles appear mostly in a concentration range of between the presence of micellar solutions and hexagonal structures, whereas those built of continuous layers appear between concentrations determining the presence of hexagonal and lamellar phases. The cubic phases are practically identified in aqueous solutions. The data on the structures in non-aqueous solutions are fragmentary [18–21]. Due to its structure, the cubic phase is inactive under polarized light. Therefore, small angle X-ray diffraction is the main source of information.

### 2.2. Identification of Liquid Crystalline Structures

**Figure 1.**
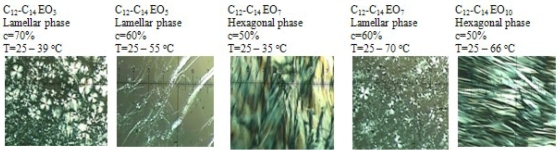
Microscopic examinations of fatty alcohols ethoxylated with 3 (70%), 5 (60%), 7 (50% and 60%), 10 (50%) moles of ethylene oxide under polarized light.

Lyotropic liquid crystals show optical changes under polarized light. If a liquid crystalline substance is placed under a microscope between crossed polarizers, rotation of polarization plane occurs. Various patterns in diverse colours (texture) can be observed in the image obtained. The texture is different for different phases. The cubic phase is optically isotropic and appears black under a polarizing microscope. Hexagonal structures give fan-like structures while the lamellar ones give “strip-like” structures (Figure 1).

Polarizing microscopy is a fast method used to assess the conditions (temperature, pressure) under which mesophases appear. In practice, the examination is carried out in such a way that pure constituents of the system being examined or solutions of a given composition are placed on a microscopic slide in such a way that they cannot come into contact with each other. The preparation is then covered with a cover slip in order to prevent evaporation of the solvent. Gradient of concentration of individual constituents is formed due to diffusion. Formation of various mesophases, which differ in texture, can be observed. Microscopic observations cannot be the only assessment criterion for lyotropic liquid crystals. They should be verified using other methods.

The measurements were conducted at 25 °C for a wide range of concentrations from 10% to nearly 100%. The effect of temperature on the formation of liquid crystals was analysed for some selected concentrations at which the presence of mesophases was discovered and the studies were carried out over a temperature range from 25 °C to disappearance of the structures.

Aqueous solutions of four alcohol ethoxylates with C_12_-C_14_ alkyl chains and different ethoxylation degrees (C_12_-C_14_ EO_m_, where m = 3, 5, 7, 10) were selected as potential lubricating substances in a liquid crystalline form. The criterion for the selection of compounds to be used in tribological studies was the presence of anisotropic phases (hexagonal or lamellar) in aqueous solutions of the compounds. The behaviour of these types of lubricants might be compared to the behaviour of graphite and molybdenum disulfide in the friction zone. The two compounds have a hexagonal structure and weaker bonds between their atoms perpendicular to lattice planes. As a result of preliminary studies, several compounds which can form mesophases were selected.

The presence of mesophases is also temperature-dependent. Therefore, microscope pictures were taken for each solution exhibiting liquid crystalline properties in a temperature range from 25 °C to disappearance of the mesophase. The analysis of the data obtained makes it possible to determine concentration and temperature intervals at which mesophases appear (Figure 1).

### 2.3. The Viscosity of Solutions Forming Liquid Crystalline Structures

Viscosity is an important determinant of the quality of a lubricating substance and it determines friction conditions. Therefore, its determination is essential from a tribological point of view. The measurements of the coefficient of dynamic viscosity (η) aimed also at confirmation of the presence of liquid crystalline structures in solutions. The hexagonal phase exhibits a high degree of packing of cylindrical micelles and, as a result, by high values of the coefficient of viscosity. The lamellar phase shows significantly lower η values which can be explained by relatively easy displacement of layers (lamellae). The identification of the relative displacement of crystallographic planes of LLCs (slip planes) is complicated. A number of premises indicate that slip planes appear at the interface of the aqueous phase and ordered surfactant molecules [11,22].

**Figure 2.**
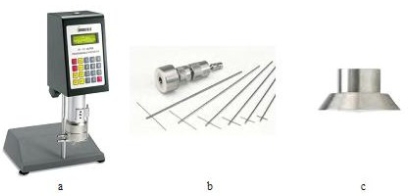
The Brookfield HADV-III Ultra viscometer, a–device, b–set of Helipath spindles, c–spindle of the CPE-52 cone type.

The Brookfield HADV-III Ultra (Figure 2a) viscometer with a set of Helipath spindles (Figure 2b) and a spindle of the CPE-52 cone type (Figure 2c) was used to measure the coefficient of dynamic viscosity of aqueous solutions of ethoxylates.

Two independent test methods were used to determine:

- the effect of concentration of additives on viscosity coefficient of solutions at a constant temperature (25 °C). The measurements were conducted using a Helipath type spindle (Figure 2b) at four rotational speeds: 5, 10, 50, 100 rpm. The concentrations of the compounds were from 0 to 100% at every 10%;
- the dependence of viscosity coefficient on shear rate from 0 to 500 s^−1^. The measurements were carried out for a cone-plate system (Figure 2c) at three temperatures: 25, 40 and 55 °C and for those solutions in which mesophases were found.

#### 2.3.1. Changes in Viscosity Coefficient as a Function of Concentration of Additives

**Figure 3.**
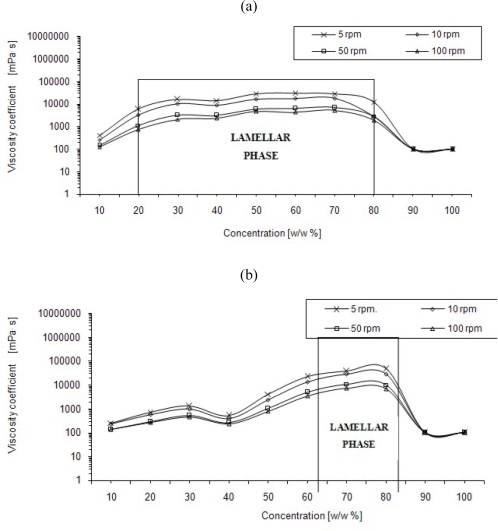

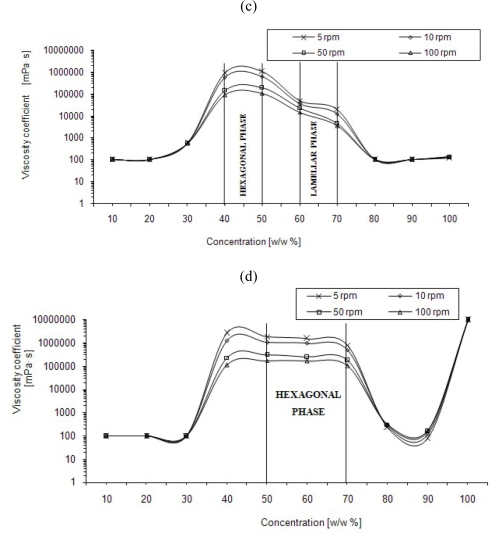
Dynamic viscosity coefficients (η) of aqueous solutions of four fatty alcohols ethoxylated with 3 (a), 5 (b), 7 (c) and 10 (d) moles of ethylene oxide. Brookfield HADV-III Ultra viscometer, Helipath spindles, rotational speed of spindles: 5, 10, 50, 100 rpm, temperature of the measurements 25 °C.

Dynamic viscosity coefficients (η) of aqueous solutions of four fatty alcohols ethoxylated with 3, 5, 7 and 10 moles of ethylene oxide were determined and denoted as C_12_-C_14_ EO_3_, C_12_-C_14_ EO_5_, C_12_-C_14_ EO_7_, C_12_-C_14_ EO_10_ (Figures 3 a–d).

A characteristic feature of C_12_-C_14_ EO_3_ solutions is a wide concentration interval (20–80%) at which the lamellar phase appears (Figure 3a). The coefficients of viscosity have values from about twenty thousand to over 30,000 mPa·s. These results correspond well with literature data [3,23,24]. A significant drop in viscosity (nearly eight-fold) is observed with an increase in rotational speed from 5 to 100 rpm over a concentration range at which the lamellar phase appears. The effect of rotational speed on viscosity observed for C_12_-C_14_ EO_3_ solutions at a constant temperature is an additional confirmation of the presence of mesophases identified by means of microscopy under polarized light. This rule applies to all the ethoxylate solutions tested.

The viscosity of solutions C_12_-C_14_ EO_5_ (Figure 3b) measured at a rotational speed of 5 rpm increases from 250 mPa·s to 1 300 mPa·s over a concentration range from 10 to 40%. With over 40% of an additive the rate of increase in the η value is higher and a maximum occurs at a concentration of 80% and amounts to about 48,000 mPa·s. A sudden viscosity drop to about 100 mPa·s is observed for the 90% solution and the pure compound (100%). Increasing the rotational speed of the spindle from 5 rpm to 100 rpm results in a several-fold drop in viscosity in the solutions in which the lamellar phase is formed (60–80%). As an example: the viscosity of the 80% solution was 48,000 mPa·s at 5 rpm, whereas at 100 rpm it decreased to a value of 7,000 mPa·s. This decrease is significantly lower or no changes can be observed over a range of both lower and higher concentrations. Viscosity values for the 60 to 80% concentration range solutions were from 20,000 do 50,000 mPa·s which is characteristic of lamellar phases. Literature data and microscopic examination results confirm the presence of lamellar structures. The dependence of viscosity on concentration of aqueous C_12_-C_14_ EO_7_ solutions can be seen in Figure 3c

The dependence of the coefficient of viscosity on concentration is not monotonic (Figure 3c). A distinct maximum can be observed over a range of concentrations for which the appearance of the hexagonal phase has been confirmed. The η value at a maximum exceeds 10^6^ mPa·s. As expected, the lamellar phase (60–70%) shows significantly lower values—about 19,000 mPa·s. The influence of the rotational speed of the spindle can be observed predominantly in the 40 to 70% concentration range. An increase in the rotational speed results in a significant drop in viscosity. With the rotational speed being increased from 5 rpm to 100 rpm, one can observe even about a ten-fold decrease in the value of η for the hexagonal phase and over a three-fold decrease for the lamellar phase.

The dependence of the coefficient of dynamic viscosity (η) on concentration of a fatty alcohol ethoxylated with 10 moles of ethylene oxide (C_12_-C_14_ EO_10_) has been shown in Figure 3d.

The dependence η (c) is characterized by a wide concentration range (40–70%) for which viscosity coefficient values are high (Figure 3d) and that range exceeds the concentration interval at which the hexagonal phase appears (50–70%). Viscosity reaches the value of about 2.7 × 10^6^ mPa·s at the concentration of 40%. One can formulate a hypothesis that at that concentration a highly viscous cubic phase is formed and it is not active under polarized light. The viscosity of the hexagonal phase (50–70%) is as expected and it ranges from 800,000 do 1,800,000 mPa·s. A significant drop in viscosity can be observed at low (c < 40%) and high (c > 70%) concentrations. “Pure” C_12_-C_14_ EO_10_ is practically a solid; therefore, viscosity was not measured in this case, and the boundary measurement value was conventionally assumed for the device used (Figure 3d). A significant drop in viscosity can be observed with an increase in rotational speed in the 40 to 70% concentration range. An increase in rotational speed from 5 rpm to 100 rpm brings about over a twenty-fold drop in the η value at the 40% concentration and about a ten-fold drop at the concentrations for which hexagonal phases appeared (50–70%). This is another confirmation of the presence of liquid crystalline structures.

#### 2.3.2. Changes in Viscosity as a Function of Shear rate

**Figure 4.**
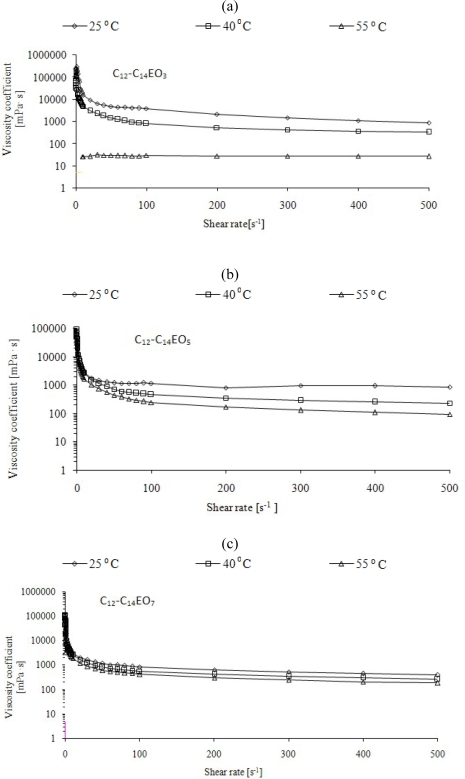

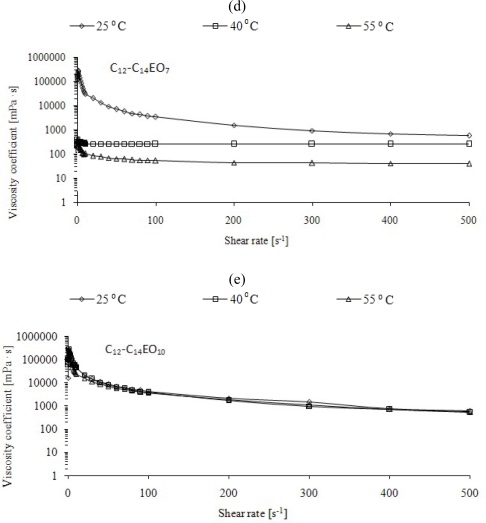
Dynamic viscosity (η(ν)) of aqueous solutions of ethoxylated fatty alcohols. Brookfield HADV-III Ultra viscometer, CPE-52 spindle, shear rate 0.02÷500 s^−1^, temperatures of the measurements 25, 40 and 55 °C.

The influence of rotational speed (Figures 3a–d) on a viscosity coefficient value confirmed the presence of mesophases which were identified by means of microscopy under polarized light. Measurements of dynamic viscosity as a function of shear rate [η(ν)] at three temperatures 25, 40 and 55 °C were a continuation of this research direction. In the case when viscosity changes significantly as a function of shear rate, the presence of mesophases should be taken into account. Figures 4a–e shows changes in the coefficient of viscosity as a function of shear rate for 50–70% solutions of ethoxylates at which liquid crystalline structures were formed.

The results obtained correspond well with the results of microscopic examinations under polarized light (Figure 1) and with the measurements of viscosity as a function of concentrations of compounds (Figure 3). The solutions forming liquid crystalline structures exhibit significant changes in η (ν). These are solutions of ethoxylates: C_12_-C_14_ EO_5_ (lamellar phase), C_12_-C_14_ EO_7_ (lamellar phase), whereas in the case of C_12_-C_14_ EO_3_ solutions the lamellar phase disappears at about 40 °C and no changes in viscosity as a function of temperature can be observed over that temperature. No significant changes can be observed as a function of shear rate also for a 50% solution of alcohol ethoxylated with seven moles of ethylene oxide (C_12_-C_14_ EO_7_) which forms a hexagonal phase at 35 °C while an isotropic liquid appears at 40 °C and 55 °C.

The results obtained indicate that liquid crystals behave like non-Newtonian fluids. They exhibit high viscosity which changes due to external forcings which lead to a modification of the structures or to their ordering in such a way that the internal resistance of the liquid decreases. It is different in the case of an isotropic liquid which behaves like a Newtonian fluid and its coefficient of viscosity does not change. The dependences η(ν) for solutions of ethoxylated alcohols will be interpreted on the basis of these assumptions.

### 2.4. Antiseizure Properties of Aqueous Solutions of Alcohol Ethoxylates Forming Mesophases

Methods of measuring antiseizure properties have been described in literature [25–27]. The aim of this study is to determine a correlation of the moment of friction force (M_T_) as a function of load (P) increasing at a rate of 409 N/s. The quantities measured were: scuffing load (P_t_), seizure load (P_oz_).

Scuffing load (P_t_) is the lowest load at which a significant increase in the moment of friction force is observed. Exceeding the value of P_t_ indicates that the lubricating layer has been broken. The P_t_ value determines stability of the lubricating film.

At seizure load (P_oz_) the spindle stops and the moment of friction force exceeds 10Nm. The Poz value describes the maximum force which can be applied to the system without destroying it.

Limiting pressure of seizure (p_oz_) describes pressures present in the tribosystem at seizure load (P_oz_).

**Figure 5.**
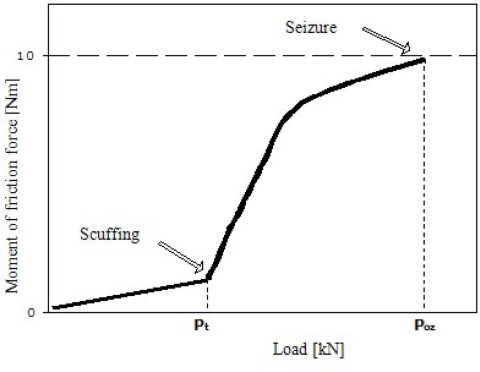
Model dependence of moment of friction force (M_T_) as a function of constant load (P) increase.

Interpretation of results obtained is depicted in Figure 5.

Limiting pressure of seizure (p_oz_), which was calculated according to the equation:

$$p_{oz}=0.52\;\cdot \;\frac{P_{oz}}{d^{2}}$$

where: P_oz_ – seizure load, d – wear scar diameter measured after the test. p_oz_ is expressed in N·mm^−2^, P_oz_ in N, and d in mm.

**Figure 6.**
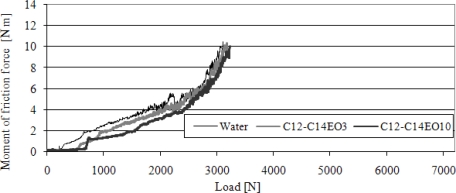
The course of changes in moment of friction force (M_T_) as a function of load (P) for water and 50% solutions of alcohols ethoxylated with 3 and 10 moles of ethylene oxide (C_12_-C_14_ EO_3_ and C_12_-C_14_ EO_10_). Four-ball tester, rotational speed of spindle 500 rpm.

The course of changes in M_T_ (P) for water and 50% solutions of alcohols ethoxylated with three and 10 moles of ethylene oxide (C_12_-C_14_ EO_3_ i C_12_-C_14_ EO_10_) has been shown in Figure 6.

**Figure 7.**
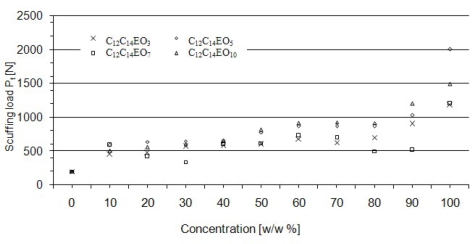
The dependence of scuffing load on concentration of aqueous solutions of alcohols ethoxylated with 3–C_12_-C_14_ EO_3_, 5–C_12_-C_14_ EO_5_, 7–C_12_-C_14_ EO_7_, 10–C_12_-C_14_ EO_10_ moles of ethylene oxide. Four-ball tester, rotational speed of spindle 200 rpm, load increasing rate 409 N/s.

In the initial phase of the test the moment of friction force increases slightly until load takes the value of scuffing load (P_t_). Above that point, M_T_ increases sharply up to 10 N·m, and load takes the value of seizure load (P_oz_). Compared to micellar solutions, there is a characteristic low, practically close to zero, increase in the M_T_ value in relation to the P_t_ value. The dependence of scuffing load on concentration of aqueous solutions of alcohols C_12_-C_14_ EO_3_, C_12_-C_14_ EO_5_, C_12_-C_14_ EO_7_, C_12_-C_14_ EO_10_ has been presented in Figure 7.

The P_t_ value obtained for water is 200 N. Addition of an amphiphilic compound results in an increase in scuffing load. In the case of C_12_-C_14_ EO_3_ solutions, P_t_ increased from 450 N for 10% solutions to 1,200 N for a pure compound (100%). An analogous dependence was observed for C_12_-C_14_ EO_5_ and C_12_-C_14_ EO_10_ solutions. P_t_ increased from 600 N to 2,000 N for C_12_-C_14_ EO_5_ and from 510 N to 1,500 N for C_12_-C_14_ EO_10_ over the whole range of concentrations studied. A slightly different course can be observed in the case of the curve of the dependence of scuffing load on concentration of C_12_-C_14_ EO_7_ solutions. In the 10 to 90% concentration range the value of P_t_ ranges from 350 N to 700 N while for a pure compound it is 1,200 N. A significant increase in scuffing load can be observed and it increases as much as 5-fold in relation to water.

**Figure 8.**
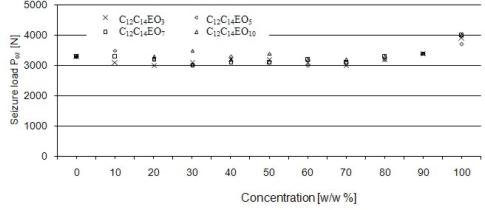
The effect of concentration of an amphiphilic compound on seizure load for aqueous solutions of alcohols ethoxylated with 3–C_12_-C_14_ EO_3_, 5–C_12_-C_14_ EO_5_, 7–C_12_-C_14_ EO_7_, 10–C_12_-C_14_ EO_10_ moles of ethylene oxide. Four-ball tester, rotational speed of spindle 200 rpm, load increasing rate 409 N/s.

The effect of concentration of an amphiphilic compound on seizure load for aqueous solutions of alcohols has been shown in Figure 8.

**Figure 9.**
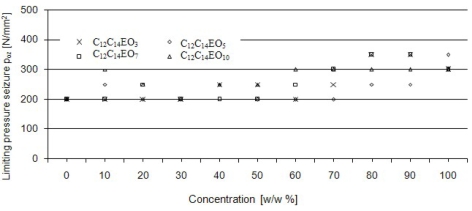
The effect of concentration on the value of limiting pressure of seizure (p_oz_) of aqueous solutions of alcohols ethoxylated with 3–C_12_-C_14_ EO_3_, 5–C_12_-C_14_ EO_5_, 7–C_12_-C_14_ EO_7_, 10–C_12_-C_14_ EO_10_ moles of ethylene oxide. Four-ball tester, rotational speed of spindle 200 rpm, load increasing rate 409 N/s.

Seizure load P_oz_ takes the values between 3,000 and 4,000 N for all solutions of fatty alcohol ethoxylates. No effect of concentration on the value of limiting pressure of seizure has been observed practically in the whole range of concentrations of aqueous solutions of alcohols examined (Figure 9). The limiting pressure of seizure values range from 200 to 350 N/mm^2^.

It appears from the analysis of the quantities characterizing seizure properties that ethoxylated compounds, including those that form liquid crystalline structures, have the strongest influence on scuffing load P_t_. The P_t_ values for solutions are several times higher than those measured for water. On average, ethoxylates with a higher degree of ethoxylation have higher values of scuffing load. These results can be interpreted in terms of dehydration. Ethoxylates with lower values of *m* have lower cloud point values, so they lose their amphiphilic properties at lower temperatures. Seizure load (P_oz_) and limiting pressure of seizure (p_oz_) take comparable values for solutions of various concentrations. So, the action of compounds or mesomorphic structures in the friction zone probably consists in formation of a film protecting friction pairs against seizure. Alcohol ethoxylates do not react chemically with a steel surface which would result in formation of wear products protecting the system against seizure. Their behaviour may be beneficial under moderate friction conditions and in those applications where chemical wear should be avoided.

## 3. Summary

The choice of ethoxylated lauryl alcohols was important, as their properties, including formation of liquid crystalline structures, have at length been described in literature, so it is possible to relate one’s own test results to literature data and confirm the former. The hydrophobic part is a permanent part of ethoxylated lauryl alcohols. It is composed of alkyl chains consisting of 12 and 14 carbon atoms connected by means of saturated bonds due to which the compound exhibits relatively high resistance to oxidation. The hydrophilic part is variable and it consists of an ethylene oxide chain with 3, 5, 7, and 10 ethylene oxide molecules per 1 alcohol molecule. Water solubility increases and surface activity decreases with an increase in ethoxylation degree. Mesophases appear in alcohol solutions over various temperature and concentration ranges. Solutions C_12_-C_14_ EO_3_ generate a lamellar phase over a wide concentration range from 20 to 80%. Solutions C_12_-C_14_ EO_3_ and C_12_-C_14_ EO_5_ form lamellar phases with viscosity reaching several dozen thousand mPa·s, whereas solutions C_12_-C_14_ EO_7_ and C_12_-C_14_ EO_10_ form hexagonal phases whose viscosity ranges from a few hundred thousand to 2 million mPa·s. The solutions lost their liquid crystalline properties at higher temperatures: 70% C_12_-C_14_ EO_3_ at 38 °C (lamellar phase), 50% C_12_-C_14_ EO_5_ at 33 °C (hexagonal phase), 60% C_12_-C_14_ EO_5_ at 78 °C (lamellar phase), 70% C_12_-C_14_ EO_7_ at 69 °C (lamellar phase). Above those temperatures the solutions were isotropic. Apart from identifying liquid crystalline structures by means of microscopy under polarized light, their presence was confirmed by rheological measurements. The viscosity coefficient values are characteristic for individual phases and the values of η decrease as a function of increasing shear rate (ν) and temperature. A drop in the value of η with an increase in shear rate, characteristic of non-Newtonian fluids, and transition to a Newtonian fluid at a given temperature were found. The results obtained for ethoxylated alcohol solutions and the conclusions drawn on their basis correspond well with literature data. This is particularly interesting, because the alcohols used in these test are produced on an industrial scale from plant raw materials and may differ from alcohols of defined purity obtained from other raw materials. Solutions of a required viscosity can be obtained by appropriate choice of the type and concentration of compound and the type of mesophase. Thus, alcohols can be used as viscosity modifiers in lubricating substances.

On the basis of the quantities P_t,_ P_oz,_ p_oz_ determined, it may be postulated that a stable lubricating film forms under friction conditions in the presence of additives able to form mesophases and that the film effectively separates co-operating friction pair elements up to a load which is equal to scuffing load. The probable causes are high surface activity and formation of ordered structures. Due to low chemical reactivity of ethoxylates with steel surfaces, wear products are not generated. Those products would result in a considerable increase in seizure load (P_oz,_) and in limiting pressure of seizure (p_oz_). If this hypothesis were valid, the compounds used would exhibit limited tribochemical wear as additives. Based on the seizure test results it may be stated that ethoxylate solutions capable of forming anisotropic LLCs show very good load-carrying capacity up to about 2.0 kN.

On the basis of the test results obtained it may be stated that ethoxylates of the fatty alcohols used are effective additives which may favourably modify lubricating properties of water and shape rheological properties of these model lubricating substances. Physicochemical properties of aqueous solutions of ethoxylates cannot be connected with their tribological characteristics in each of the dependences obtained. However, a number of experimental facts point to a role of surface activity of the compounds and an effect of lyotropic liquid crystals on antiseizure properties. Measurements of resistance to motion and wear at constant loads are a continuation of the studies on antiseizure capabilities. The studies were carried out for various loads and also for various materials and geometries of friction pairs. The results obtained will be published.
